# Tissue Doppler imaging of the diaphragm and outcome of weaning from mechanical ventilation

**DOI:** 10.1002/ajum.12389

**Published:** 2024-05-06

**Authors:** Shaobo Xin, Yingjia Li, Rui Liu, Xiaozhen Liu, Shaoqing Cai

**Affiliations:** ^1^ Department of Medical Ultrasonics, Nanfang Hospital Southern Medical University No. 1023, South Shatai Road, Baiyun District Guangzhou 510515 China; ^2^ Intensive Care Unit Zhongshan City People's Hospital No. 2, Sunwen East Road Zhongshan Guangdong Province 528403 China; ^3^ Department of Medical Ultrasonics Zhongshan City People's Hospital No. 2, Sunwen East Road Zhongshan Guangdong Province 528403 China

**Keywords:** diaphragm ultrasound, tissue Doppler imaging technique, mechanical ventilation weaning prediction

## Abstract

**Purpose:**

This study aimed to employ tissue Doppler imaging to monitor diaphragmatic peak velocity and acceleration during contraction and relaxation in mechanically ventilated patients, with the objective of assessing the potential utility of this technique in predicting weaning outcomes.

**Methods:**

A total of 89 adult subjects were recruited in this study. After 30 min of spontaneous breathing trial, the diaphragm motion parameters, including peak contraction velocity, peak relaxation velocity, contraction acceleration and relaxation acceleration, were measured in real time using tissue Doppler imaging. According to the results of weaning, the patients were divided into successful weaning group and failed weaning group. The differences of diaphragmatic tissue Doppler imaging monitoring indicators between the two groups were analysed, and the receiver operating characteristic curve was drawn to analyse the value of each ultrasound parameter in predicting weaning.

**Results:**

In the successful weaning group, there were 61 subjects, while in the failed weaning group, there were 28 subjects. The peak contraction velocity, peak relaxation velocity, contraction acceleration and relaxation acceleration of the diaphragm were significantly higher in the failed weaning group compared to the successful weaning group (P < 0.05). The area under the curve of diaphragmatic peak contraction velocity, peak relaxation velocity, diaphragmatic contraction acceleration and diaphragmatic relaxation acceleration were 0.81 (0.72–0.91), 0.85 (0.77–0.93), 0.74 (0.63–0.86) and 0.86 (0.78–0.94), respectively.

**Conclusions:**

The diaphragm ultrasonic tissue Doppler imaging variables can serve as predictive indicators for weaning mechanical ventilation in patients, thus providing an effective tool to assist critical care physicians in determining the optimal timing for weaning mechanical ventilation.

## Introduction

Mechanical ventilation (MV) represents a pivotal life support modality in intensive care units (ICUs). Globally, approximately 15 million patients receive MV annually.^1^ In China, approximately 70% of patients admitted to ICU undergo MV.^2^ Despite successful weaning from ventilators in many critically ill patients, approximately 30% of patients still experience weaning failure.^3^ If patients undergoing MV are prematurely weaned, it may result in increased cardiovascular and respiratory system pressure, further exacerbating carbon dioxide retention and hypoxemia, leading to re‐intubation and an elevated risk of mortality.^4^ Prolonged MV can give rise to associated complications such as ventilator‐associated pneumonia, airway injury, among others, resulting in substantial healthcare expenses.^5^ Therefore, reaching a precise determination of the optimal timing for successful weaning is of utmost importance.

The diaphragm serves as a vital inspiratory muscle, responsible for approximately 80% of the work involved in respiration. Consequently, the function of the diaphragm plays a pivotal role in ventilation. Excessive MV can lead to disuse muscle atrophy, resulting in prolonged duration of MV.^6^ Conversely, inadequate ventilatory support may prevent sufficient rest for the diaphragm and consequently cause overload injury to this muscle.^7^ These aforementioned factors contribute to and progressively exacerbate dysfunction of the diaphragm, ultimately leading to ventilator‐induced diaphragmatic dysfunction (VIDD).^8^

Currently employed prediction parameters for offline ventilator use lack effectiveness in practical application. For instance, although the rapid shallow breathing index (RSBI) is readily obtainable and widely utilised in clinical practice during the early stages, there exists no standardised reference threshold,^9^ making it not feasible to directly monitor patients' diaphragm movement or assess their diaphragm function. Currently, various techniques are available for assessing diaphragmatic function, including X‐ray, CT, MRI, transdiaphragmatic pressure (Pdi) measurement and detection of diaphragmatic electrical activity. Although these methods play a certain role in diaphragm evaluation, they are not deemed suitable for examining patients in the ICU.^10^, ^11^ Ultrasound examination possesses the attributes of portability, non‐invasiveness, real‐time dynamic observation capability, cost‐effectiveness, and absence of radiation. Consequently, it has gained extensive utilization in ICU.^12^ However, conflicting findings have been reported in studies assessing diaphragm contraction velocity using conventional ultrasound as a predictor of successful weaning,^13^, ^14^ and there is currently a lack of systematic investigation into the role of diaphragm kinetic parameters in predicting successful weaning. In comparison to conventional ultrasound technology, ultrasound tissue Doppler imaging (TDI) enables real‐time monitoring of velocity changes in moving tissues and facilitates quantitative analysis, thereby enabling more accurate evaluation of the diaphragm's motion state. In echocardiography, TDI‐derived peak contraction velocity of the myocardium exhibits correlation with ejection fraction and serves as an identifier for myocardial systolic dysfunction. Therefore, we hypothesized that the diaphragm's TDI variables (dTDI) may serve as a supplementary indicator for predicting the outcome of weaning in patients undergoing MV.

We conducted a prospective observational study to compare dTDI variables in mechanically ventilated patients during successful or unsuccessful weaning after 30 min of spontaneous breathing trial (SBT). To assess the potential of dTDI variables in predicting weaning outcomes among mechanically ventilated patients.

## Materials and methods

### Participants

A total of 89 patients, admitted to the medical ICU of Zhongshan City People's Hospital between April 2023 and October 2023, who underwent MV for more than 48 h and were eligible for weaning, were included in this study. Following the commonly employed weaning protocol at our treatment centre, the pressure support ventilation (PSV) mode was selected as the SBT mode. Inclusion criteria comprised: (i) age ≥ 18 years; (ii) MV duration exceeding 48 h; (iii) refractory respiratory failure resulting from diverse aetiologies; (iv) patients who fulfilled the weaning criteria for SBT, such as oxygenation index exceeding 150 mmHg; positive end‐expiratory pressure (PEEP) below 5 cmH_2_O; autonomous and forceful ability to cough; discontinuation of sedatives and vasopressors.^15^ Exclusion criteria encompassed: (i) age < 18 years; (ii) prior lung tumour or resection, chest wall deformity or abnormal respiratory mechanics; (iii) Neuromuscular junction‐related disorders or diaphragmatic paralysis; (iv) repression of respiratory central function resulting from spinal cord injury.

### Methods

The intensivist regularly monitored mechanically ventilated patients in the intensive care unit and conducted SBT on eligible patients who meet the inclusion criteria. If the patient successfully completed a 30‐min SBT, bedside ultrasound was utilised to evaluate dTDI variables and other conventional diaphragmatic ultrasound variables (including diaphragmatic excursion and inspiratory time). If the patient exhibited the following symptoms within a 30‐min timeframe during the spontaneous breathing trial, including: excessive perspiration, irritability and lethargy; peripheral blood arterial oxygen saturation decreased by ≥5%;respiratory rate exceeded 35 beats/min or showed an increase of more than 50% compared to the pre‐SBT level; systolic blood pressure exhibited a rise of over 20%; heart rate surpassed 140 beats/min or demonstrated an increment of more than 20% from the pre‐SBT level, immediate cessation of the spontaneous breathing test was warranted and mechanical ventilation was reinstated.^16^ Following completion of the 30‐min SBT, patients underwent a weaning process, and based on the weaning outcomes observed after 48 hours, they were categorised into successful and unsuccessful weaning groups.

To guarantee the precision and dependability of the data, two experienced sonographers were engaged in the data collection process to eliminate potential biases among diverse participants. The two sonographers possessed extensive expertise in ultrasound with over a decade of professional experience, demonstrating requisite knowledge and proficiency. Each sonographer was capable of independently conducting bedside ultrasonography for patients in the ICU. During the initial phase of the study, two sonographers underwent training and education in diaphragmatic ultrasonography.

### Definition of terms

Successful weaning was defined as the ability to maintain spontaneous breathing for 48 hours without re‐intubation after a successful 30‐min SBT. Weaning failure was defined as the inability to maintain spontaneous breathing, requiring reintubation or resulting in death within 48 hours of continuing weaning despite a successful 30‐min SBT.^17^

### Ultrasound examinations

The ultrasound instrument utilised in this study was a PHILIPS brand HD15 (Amsterdam, the Netherlands).

**Figure 1:**
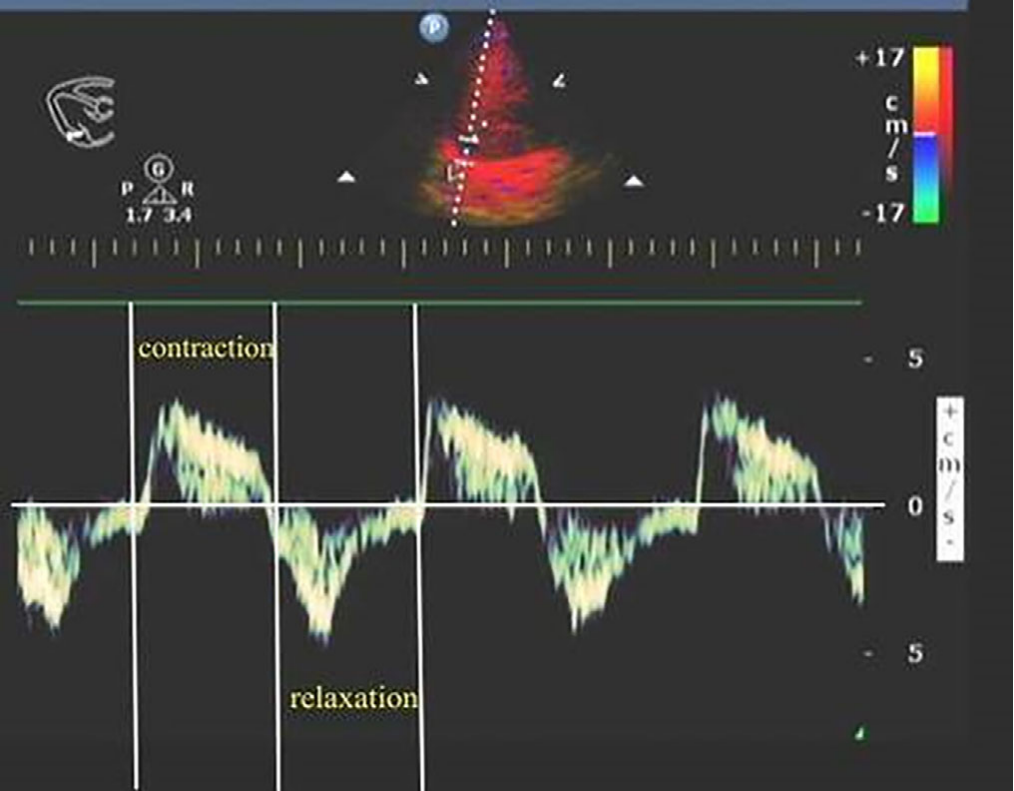
Schematic representation of tissue Doppler imaging during the contraction and relaxation of the diaphragm. dTDI exhibits two waves, one during diaphragmatic contraction (above the baseline) and one during diaphragmatic relaxation (below the baseline). dTDI, tissue Doppler imaging variables of the diaphragm.

**Figure 2:**
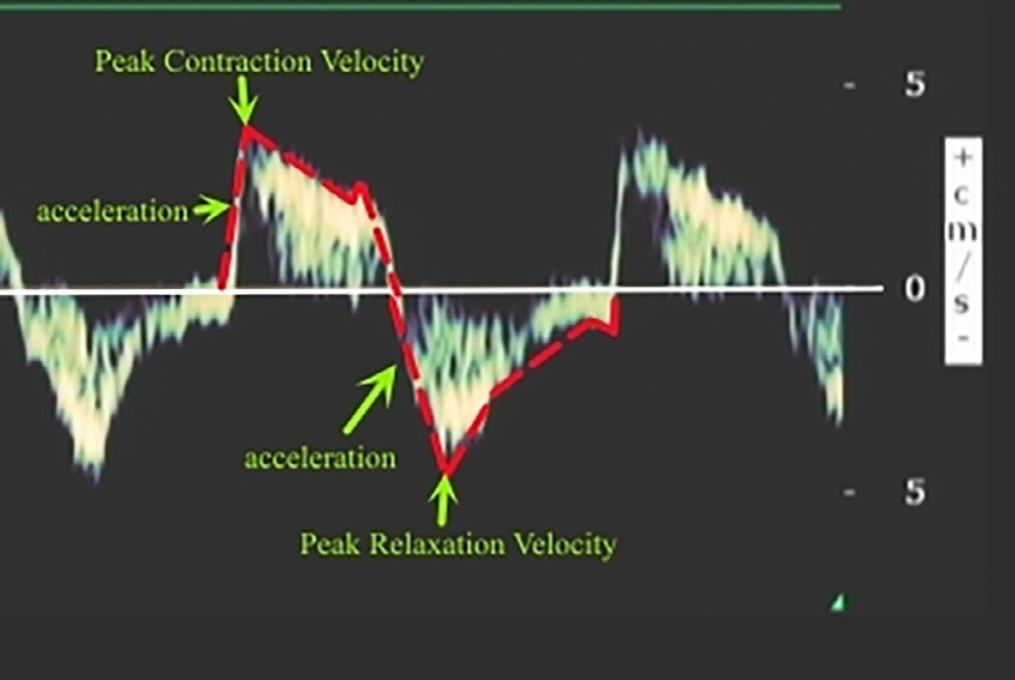
Schematic representation illustrating the measurement of diaphragm tissue Doppler imaging variables. Contraction peak velocity = the maximal velocity during contraction (cm/s); contraction acceleration = the slope of the steepest portion of the contraction velocity curve from baseline to contraction peak velocity (cm/s^2^); peak relaxation velocity = the maximum diaphragmatic velocity during relaxation (cm/s); relaxation acceleration = the slope of the steepest portion of the relaxation velocity curve from baseline to peak relaxation velocity (cm/s^2^).

The dTDI variables were obtained by placing the patient in a supine position and positioning the phased array probe (2–4 MHz) subcostally between the midclavicular line and anterior axillary line 30 minutes after starting SBT. The probe was then directed cephalad towards the lower edge of the costal arch, using the liver as a transducing window to obtain a top view of the diaphragm in its right lateral position (allowing observation of hepatic vein confluence into inferior vena cava via liver). To ensure accurate TDI measurement, sampling lines were made perpendicular to the top of the diaphragm. A sampling frame width of 18.5–20.0 mm was chosen to incorporate entire range of diaphragmatic motion due to its large movement amplitude. A velocity scale of 5 cm/s was selected. By attenuating gain and excluding high‐frequency signals, clear tissue movement velocity waveform could be obtained from which peak contraction velocity (d‐PCV) can be measured as highest point on inspiratory curve with unit cm/s. Acceleration of diaphragm contraction (d‐AC) was the slope of the steepest part of the inspiratory velocity curve from baseline to peak inspiratory velocity, in cm/s^2^. The peak relaxation velocity of the diaphragm (d‐PRV) was defined as the velocity at the nadir of the expiratory velocity curve, measured in cm/s. Meanwhile, acceleration of diaphragmatic relaxation (d‐AR) refers to the slope of the steepest segment of this same curve from baseline to peak expiratory velocity, expressed in units of cm/s^2^. Three consecutive respiratory cycles were observed and these variables were recorded during a single cycle; an average value was calculated based on three measurements (Figures 1 and 2).

### Clinical data of general nature

Additional observations included recording patients' age, gender, reason for ICU admission, ventilation duration before weaning and parameters such as patients' heart rate and mean arterial blood pressure were checked before performing SBT were recorded.

### Statistical analysis

The data were analysed using SPSS 20.0 software. Continuous variables were described as mean $\bar{X}$ ± S standard deviation, while non‐normally distributed continuous variables were described as median and interquartile range M (P25, P75). For comparisons between two groups with normally distributed information, the independent samples *t*‐test was employed; for comparisons of non‐normally distributed information, the Wilcoxon rank sum test was chosen. Count data were statistically processed using the χ^2^ test. The GraphPad Prism 8 software was utilised for generating the receiver operating characteristic (ROC) curve and calculating the area under the curve (AUC) to evaluate the predictive value of dTDI variables for successful weaning in patients with mechanical ventilation. A statistically significant difference was considered when P < 0.05.

### Ethics approval

This study has obtained approval from the Zhongshan City People's Hospital Clinical Research and Animal Experiment Ethic Committee, and the approval code is K2023‐053. Written informed consent was obtained from all subjects.

## Results

**Figure 3:**
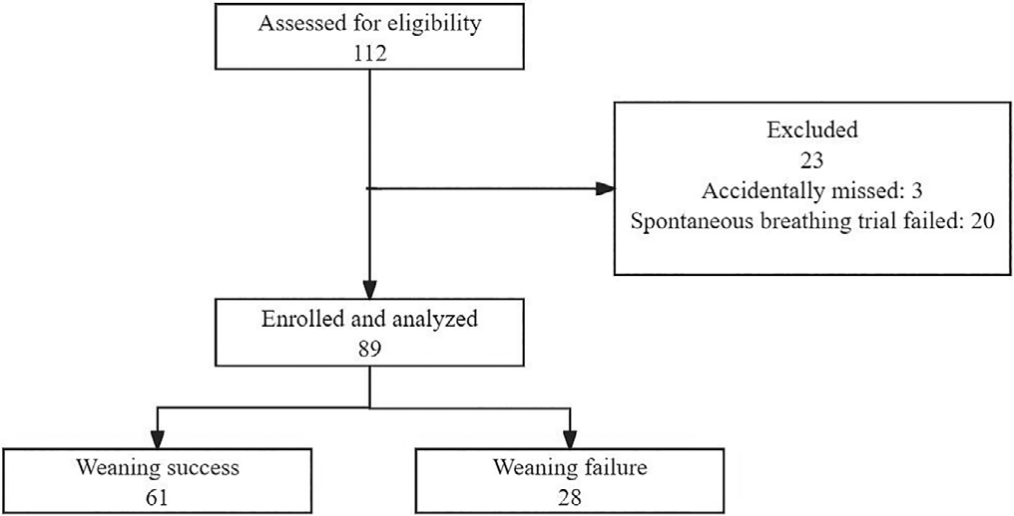
Flow chart.

**Figure 4:**
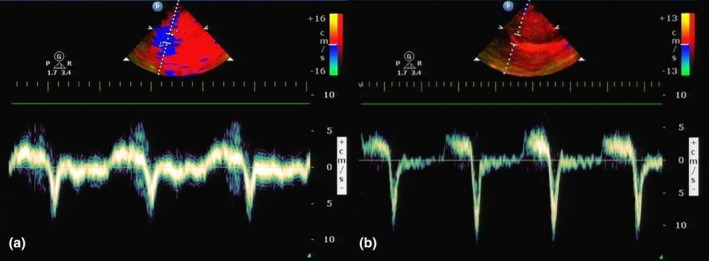
d‐TDI patterns in the two groups. (a) Patients in the successful weaning group; (b) Patients in the failure weaning group. d‐TDI, tissue Doppler imaging variables of the diaphragm.

Overall, 112 subjects were assessed for eligibility, of whom 89 subjects were enrolled and finally analysed (Figure 3). The 89 patients were included in this study, with an average age of 67.0 ± 12.7 years, comprising 65 males (73.0%) and 24 females (26.9%). Respiratory system diseases accounted for 63 cases (70.8%), cardiovascular system diseases accounted for 12 cases (13.5%), nervous system diseases accounted for 4 cases (4.5%) endocrine system diseases accounted for 4 cases (4.5%), and digestive system diseases accounted for 6 cases (6.7%). Among the enrolled patients, there were 61 cases (68.5%) in the successful weaning group and 28 cases (31.5%) in the failed weaning group. Table 1 presents comprehensive information regarding patient demographics, reason for ICU admission and vital signs prior to weaning process initiation. No significant differences were observed between the successful weaning group and the failed weaning group in terms of age, sex distribution, heart rate, tidal volume (P > 0.05). Ventilation duration before weaning and respiratory rate of the failed weaning group were significantly higher compared to those of the successful weaning group, whereas the mean arterial pressure and the inspiratory time (Ti) was significantly lower in the failed weaning group (P < 0.05). d‐MCV did not show any significant differences between the successful weaning group and the failed weaning group (P > 0.05). d‐PCV, d‐PRV, d‐CR and d‐AR acceleration were lower in the successful weaning group compared to the failed weaning group. Additionally, the successful weaning group exhibited higher levels of diaphragmatic excursion (DE) than these observed in the failed weaning group. These differences between groups were statistically significant (P < 0.05) (Table 1, Figure 4).

**Table 1:** ICU patient baseline characteristics, diaphragmatic evaluations, dTDI measurements and between‐group comparisons

	Total (n = 89)	Weaning success (n = 61)	Weaning failure (n = 28)	P values
Sex, M	65 (73.0%)	43 (70.5%)	22 (78.6%)	0.43
Age, year	67.0 ± 12.7	65.4 ± 11.7	70.5 ± 14.2	0.08
Ventilation duration before weaning, days	8 (6, 11)	8 (5, 9)	12 (9, 14)	<0.01
Reason for ICU admission				0.99
Disease of respiratory system	63 (70.8%)	43 (70.4%)	20 (71.4%)	
Diseases of the cardiovascular system	12 (13.4%)	8 (13.1%)	4 (13.3%)	
Nervous system disease	4 (4.5%)	3 (5.1%)	1 (3.6%)	
Digestive system disease	6 (6.8%)	4 (6.3%)	2 (8.1%)	
Endocrine system disease	4 (4.5%)	3 (5.1%)	1 (3.6%)	
Tidal volume, mL	307.7 ± 88.3	316.1 ± 95.2	289.3 ± 68.9	0.19
Inspiratory time, s	0.91 ± 0.29	0.99 ± 0.28	0.73 ± 0.21	<0.01
Mean arterial pressure, mmHg	85.2 ± 9.9	86.8 ± 9.5	81.9 ± 10.3	0.03
Heart rate, beats/min	86.1 ± 11.6	86.1 ± 11.3	86.2 ± 12.3	0.96
Respiratory frequency, breaths/min	19.2 ± 2.1	18.8 ± 1.8	20.0 ± 2.6	0.01
Diaphragmatic evaluations				
DE, cm	1.53 (±0.33)	1.63 ± 0.30	1.29 ± 0.29	<0.01
d‐MCV, cm/s	1.85 (±0.67)	1.80 ± 0.66	1.93 ± 0.69	0.37
dTDI measurements				
d‐PCV, cm/s	2.65 (2.24, 3.33)	2.40 (2.12, 2.72)	3.17 (2.99, 3.95)	<0.01
d‐PRV, cm/s	3.39 (2.60, 4.86)	2.95 (2.16, 3.89)	5.02 (3.72, 6.35)	<0.01
d‐AC, cm/s^2^	3.39 (2.64, 4.91)	3.14 (2.48, 4.16)	4.91 (3.25, 6.46)	<0.01
d‐AR, cm/s^2^	9.05 (6.33, 11.35)	7.50 (4.86, 9.35)	12.01 (10.00, 15.63)	<0.01

d‐AC, acceleration of diaphragm contraction; d‐AR, acceleration of diaphragmatic relaxation; DE, diaphragmatic excursion; d‐MCV, mean velocity of diaphragmatic contraction; d‐PCV, peak contraction velocity of the diaphragm; d‐PRV, peak relaxation velocity of the diaphragm; dTDI, tissue Doppler imaging variables of the diaphragm; ICU, intensive care units.

**Figure 5:**
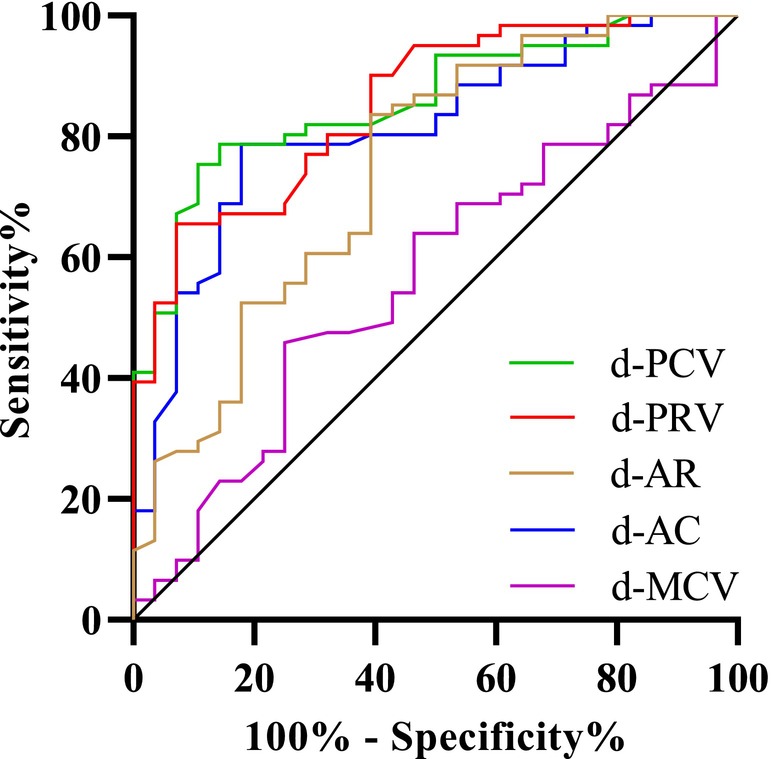
Receiver operating characteristic curves for diaphragmatic displacement tissue Doppler variables and conventional diaphragm movement velocity. d‐AC, acceleration of diaphragm contraction; d‐AR, acceleration of diaphragmatic relaxation; d‐MCV, mean velocity of diaphragmatic contraction; d‐PCV, peak contraction velocity of the diaphragm; d‐PRV, peak relaxation velocity of the diaphragm.

The ROC curve demonstrated an area under the receiver operating characteristic curve (AUC) for d‐PCV in predicting successful weaning was 0.812 (0.718–0.906), with a cut‐off value of 2.82 cm/s. The sensitivity and specificity were 82.1% and 78.7%, respectively. The positive and negative predictive values were 89.3% and 66.7%, respectively. The positive likelihood ratio and negative likelihood ratio were 3.85 and 0.23, respectively. The AUC of d‐PRV for predicting successful weaning was 0.85 (0.773–0.933), with a cut‐off value of 3.33 cm/s. The sensitivity and specificity were found to be 92.9% and 65.6%. The positive and negative predictive values were 84.8% and 78.3%, respectively. The positive likelihood ratio and negative likelihood ratio were 2.7 and 0.11, respectively. The AUC for d‐AC in predicting successful weaning was 0.74 (0.630–0.859), with a cut‐off value of 4.32 cm/s^2^. The sensitivity and specificity were determined to be 60.7% and 83.6%. The positive and negative predictive values were 88.1% and 48.9%, respectively. The positive likelihood ratio and negative likelihood ratio were 3.70 and 0.47, respectively. The AUC of d‐AR for predicting successful weaning was 0.856 (0.781–0.936), with a cut‐off value of 9.25 cm/s^2^. The sensitivity and specificity were determined to be 89.3% and 75.4%. The positive and negative predictive values were 88.5% and 75.0%, respectively. The positive likelihood ratio and negative likelihood ratio were 3.63 and 0.14, respectively. The AUC of d‐MCV for predicting successful weaning was 0.567 (0.440–0.695), with a cut‐off value of 1.56 cm/s. The sensitivity and specificity were determined to be 75.1% and 47.5%. The positive and negative predictive values were 75.4% and 46.4%, respectively. The positive likelihood ratio and negative likelihood ratio were 1.43 and 0.53, respectively (Table 2, Figure 5).

**Table 2:** Results of ROC curve plotting

	d‐PCV	d‐PRV	d‐AC	d‐AR	d‐MCV
AUC	0.812 (0.718–0.906)	0.853 (0.773–0.933)	0.744 (0.630–0.859)	0.858 (0.781–0.936)	0.567 (0.440–0.695)
Cut off value	2.82 cm/s	3.33 cm/s	4.32 cm/s^2^	9.25 cm/s^2^	1.56 cm/s
Sensitivity	82.1%	92.9%	60.7%	89.3%	75.0%
Specificity	78.7%	65.6%	83.6%	75.4%	47.5%
PPV	89.3%	84.8%	88.1%	88.5%	75.4%
NPV	66.7%	78.3%	48.9%	75%	46.4%
+LR	3.85	2.7	3.70	3.63	1.43
−LR	0.23	0.11	0.47	0.14	0.53

AUC, area under the curve; d‐AC, acceleration of diaphragm contraction; d‐AR, acceleration of diaphragmatic relaxation; d‐MCV, mean velocity of diaphragmatic contraction; d‐PCV, peak contraction velocity of the diaphragm; d‐PRV, peak relaxation velocity of the diaphragm; +LR, positive likelihood ratio; −LR, negative likelihood ratio; NPV, negative predictive value; PPV, positive predictive value; ROC, receiver operating characteristic.

## Discussion

Determining the optimal weaning time for mechanically ventilated patients remains a formidable challenge faced by critical care physicians. In the ICU, diaphragmatic muscle dysfunction in patients with severe has high morbidity. Within 24 h, over 50% of mechanically ventilated patients develop VIDD, with its incidence closely associated with prolonged mechanical ventilation, difficult weaning and reintubation rates.^18^ Despite the widespread use of diaphragmatic ultrasound for monitoring mechanical ventilation in critically ill patients, its potential role in predicting weaning failure remains a subject of controversy, and conventional ultrasound‐based measurement of diaphragmatic contraction velocity does not appear to provide a reliable reference.^19^, ^20^ The objective of this study was to employ tissue Doppler imaging for monitoring diaphragm motion in mechanically ventilated patients, and assess the potential of utilising this technology to predict weaning outcomes by acquiring variables such as peak velocity and acceleration. Additionally, it aims to establish a novel reference method for critical care physicians to accurately determine the optimal timing for weaning.

The findings of this study revealed that patients who failed to wean exhibited elevated levels of diaphragmatic kinetic parameters after 30 min of SBT, indicating a more pronounced respiratory movement compared to those who successfully weaned. d‐PCV, d‐PRV and d‐AR demonstrated the highest AUC values, demonstrating excellent sensitivity and specificity. In our study, we observed that weaning success was not achieved by any patient when d‐PCV ≥ 4.4 cm/s or d‐PRV ≥ 7.3 cm/s.

The higher d‐PCV observed in the failed weaning group may be attributed to the fact that a significant majority of patients who failed to wean experience an augmented respiratory motor output during progressive respiratory failure. The increase in minute ventilation demand often prompted patients to adjust their respiratory response by augmenting either the frequency or depth of breathing, so as to accommodate the change in respiratory pressure load.^21^ Another study conducted by Easton *et al* highlighted that patients who experienced failed weaning exhibited significantly higher respiratory load, particularly airway resistance, compared to those who successfully weaned.^22^ Additionally, the diaphragm required increased strenuous activity to ensure adequate alveolar ventilation and gas exchange.

The higher d‐PRV and d‐AR observed in patients with failed weaning may be attributed to the necessity for rapid restoration of the diaphragm's initial length, thereby preserving its capacity to generate pressure. Delayed relaxation of the diaphragm resulted in incomplete expiration of functional residual capacity, consequently shortening subsequent inspiratory processes during respiratory movements.^23^ The diaphragmatic muscle fibres, classified as skeletal muscles, primarily undergo blood perfusion during muscle relaxation. Sufficient blood supply facilitates the nourishment of the diaphragm and elimination of metabolic waste, thereby preventing muscle fatigue and maintaining optimal diaphragmatic function.^24^ The rapid relaxation of the diaphragm may serve as a compensatory mechanism to counteract impending muscle fatigue. Therefore, ensuring prompt recovery of the diaphragm to its optimal length is crucial for maintaining sufficient diaphragmatic function, particularly during periods of increased respiratory demand.

The diaphragm predominantly consists of type I muscle fibres, characterised by their slow contraction speed and lower tension production, yet exhibiting excellent resistance to fatigue. Conversely, the contractile capacity of the diaphragm is primarily determined by a smaller proportion of type II fast muscle fibres, which possess limited content and lack anti‐fatigue properties.^25^ Therefore, slow and low‐intensity exercise is the optimal mode of exercise for the diaphragm. After weaning off those patients who still had a higher respiratory load, their diaphragm was engaged in vigorous exercise. When the diaphragm exhibits intolerance, fatigue or dyskinesis may ensue^26^ particularly in patients with impaired diaphragmatic function, leading to respiratory distress or dyspnoea and ultimately resulting in unsuccessful weaning.

In the present study, there was no statistically significant difference in d‐MCV observed between patients in the failed weaning group and those in the successful weaning group. This finding aligns with a previous study conducted by Palkar *et al*.^14^ The potential reason for this disparity could be attributed to the fact that patients in the unsuccessful weaning group may exhibit diminished DE and shorter Ti concurrently compared to those in the successful weaning group. The values calculated based on the equation ‘d‐MCV = DE/Ti’ did not exhibit a discernible trend. Therefore, the d‐TDI variable demonstrates superior accuracy in predicting weaning outcomes in mechanically ventilated patients compared to d‐MCV based on conventional ultrasound measurements.

In clinical practice, special attention should be given to patients who successfully pass SBT but exhibit elevated d‐TDI variables. Close monitoring within 48 h after weaning is crucial in promptly identifying the occurrence of weaning failure and providing timely intervention.

The limitations of this study are as follows: firstly, it is important to note that this study was conducted at a single centre and had a limited number of cases. Therefore, further investigation with a larger sample size is necessary to fully explore the potential of d‐TDI variables in predicting the outcome of weaning from MV. Secondly, weaning failure in ICU patients is a complex and multifaceted issue, with potential variations in d‐TDI patterns observed between those who fail due to respiratory muscle fatigue and those who fail due to impaired respiratory drive. Thirdly, we exclusively monitored d‐TDI variables in patients who successfully completed SBT, while no monitoring observations were conducted for patients who either did not undergo SBT or failed to pass it. Furthermore, our study exclusively employed SBT in PSV mode, thus our findings may not be applicable to other modes of SBT.

## Conclusion

After a 30‐min SBT, the utilisation of TDI to measure diaphragm dynamics parameters proves valuable in identifying the risk of weaning failure among mechanically ventilated patients. d‐TDI variables exhibit significant predictive value for successfully weaning patients from MV.

## Author contributions

**Shaobo Xin:** Data curation (equal); investigation (equal); methodology (equal); project administration (equal); writing – original draft (equal). **Yingjia Li:** Writing – original draft (equal); writing – review and editing (lead). **Rui Liu:** Data curation (equal); investigation (equal); writing – original draft (equal). **Xiaozhen Liu:** Data curation (equal); investigation (equal). **Shaoqing Cai:** Data curation (equal); investigation (equal).

## Funding

This research received no specific grant from any funding agency in the public, commercial or not‐for‐profit sectors.

## Conflict of interest

All authors have disclosed no conflicts of interest.
